# Cervical disc width index is a reliable parameter and consistent in young growing Dutch Warmblood horses

**DOI:** 10.1111/vru.12913

**Published:** 2020-10-13

**Authors:** Stefanie Veraa, Carmen J.W. Scheffer, Danielle H.M. Smeets, Renske B. de Bruin, Arie C. Hoogendoorn, Johannes C.M. Vernooij, Mirjam Nielen, Willem Back

**Affiliations:** ^1^ Diagnostic Imaging, Department of Clinical Sciences, Faculty of Veterinary Medicine Utrecht University Utrecht The Netherlands; ^2^ Equine Veterinary Clinic “De Watermolen” Haaksbergen The Netherlands; ^3^ Division of Equine Sciences, Department of Clinical Sciences, Faculty of Veterinary Medicine Utrecht University Utrecht The Netherlands; ^4^ Department of Population Health Sciences, Faculty of Veterinary Medicine Utrecht University Utrecht The Netherlands; ^5^ Department of Surgery and Anaesthesiology of Domestic Animals, Faculty of Veterinary Medicine Ghent University Merelbeke Belgium

**Keywords:** disc degeneration, radiography, vertebral column

## Abstract

Intervertebral disc disease, as well as the associated alteration of the radiographic intervertebral disc space width, has been reported in horses. Disc height index (DHI) has proven to be an accurate and objective parameter in other species but data related to this parameter are lacking in horses. Therefore, the aims of this retrospective longitudinal diagnostic accuracy study were (a) to evaluate the reliability of measurements within and between observers of the equine Disc Width Index (EDWI) as a parameter for radiographic equine cervical intervertebral disc space width, and (b) to evaluate the sequential development of the EDWI over time. For this, EDWI from all intervertebral disc spaces between second cervical (C) to first thoracic (Th) vertebrae were obtained in a group of 39 Dutch Warmblood horses at 1, 5, and 18 months of age, by one European College of Veterinary Diagnostic Imaging (ECVDI) board‐certified veterinary radiologist (S.V.) and two veterinary students. Bland‐Altmann plots and intraclass Correlation Coefficient revealed a good intra‐ and interobserver agreement. A linear mixed‐effect model did reveal that mean EDWI increases significantly toward the caudal cervical spine, but did not differ significantly for a certain location over time or between sexes. Spearman's rank test did show a significant correlation between the vertebral alignment angle induced by different head‐neck positions and a normalized EDWI (*ρ* = 0.33, *P* < .0001). Student's *t*‐test revealed that the presence of C6‐C7 transposition of the transverse processes did not influence EDWI significantly. It was concluded that EDWI represents a reliable parameter for equine cervical radiographic intervertebral disc space width. Practical implementation of EDWI warrants monitoring in a group of adult horses while maintaining a standardized head‐neck position.

**Abbreviations**
Bwt: body weight
C: cervical
DHI: disc height index
DICOM: Digital Imaging and Communication in Medicine
ECVDI: European College of Veterinary Diagnostic Imaging
EDWI: equine disc width index
i.v.: intravenous
ICC: intraclass correlation coefficient
Min‐max: minimum to maximum
Th: thoracic
VAA: vertebral alignment angle.

## 1 INTRODUCTION

Intervertebral disc disease such as disc protrusion, extrusion, discospondylitis, or ischemic myelopathy due to a disc extrusion related fibrocartilaginous spinal infarct, is common in dogs and humans.^1^, ^2^, ^3^ However, it appears to be uncommon in horses, as only incidental case descriptions have been published.^4^, ^5^, ^6^, ^7^, ^8^, ^9^, ^10^ Although clinical signs such as pain, spasticity, and spinal ataxia have been reported in these cases of intervertebral disc disease, the clinical impact of intervertebral disc degeneration in the aging horse was always considered to be low.^11^ However, in more recent years cervical vertebral column pathology is increasingly recognized clinically as a source of lameness or poor performance in horses.^12^, ^13^ Degeneration of the cervical intervertebral disc has been described as disintegration of the discal fibrocartilaginous tissues with end stage cleft formation and a macroscopic degeneration,^11^ for which recently a grading system has been developed.^14^ Narrowing of the intervertebral disc space can be a feature of intervertebral disc degeneration and herniation as described in humans and several animal models, but also in horses^3^, ^15^, ^16^, ^17^, ^18^. Intervertebral disc space width has been defined and monitored by disc height calculation on lateral radiographs of the spine in humans and in several animal models.^15^, ^16^, ^17^

Morphologic variations, consisting of transposition of the transverse processes from C6 to C7, have a higher incidence in Warmblood horses^19^, ^20^ and were possibly associated with a longer C7 vertebral body length and a wider intervertebral disc space between C6 and C7.^19^ However, intra‐ and interobserver agreement for determination of the cervical intervertebral disc space width in horses has not been established yet. Measurements and evaluation of the equine disc width index (EDWI) were introduced in this study to support future research in this area of the equine neck.

Therefore, we first hypothesized that the reliability of measurements within and between observers of the EDWI as a quantitative parameter for radiographic equine cervical intervertebral disc space width from C2 to Th1 would be high. We hypothesized that the natural development of the intervertebral disc space width in a group of young, healthy horses by sequential measurements at 1, 5, and 18 months of age, would be equal to lengthening of the adjacent vertebra and hence EDWI would be consistent over time.

## 2 MATERIALS AND METHODS

### 2.1 Data collection

Archived radiographic studies of the cervical vertebral column that had been taken at the private equine veterinary clinic “De Watermolen” for breeding screening purposes of foals born between February and June 2015 were used for this retrospective observer comparison and longitudinal observational study (STARD guidelines applied). Foals were selected for breeding screening when they were 1‐month old and were radiographically examined at this time, at the time of weaning at 5 months and at 18 months when the young horses came in from the pasture. Decisions for subject inclusion or exclusion were made by a European College of Veterinary Surgery (ECVS) board‐certified equine veterinary surgeon (C.S.).

The radiographs were evaluated in a freeware DICOM viewer (RadiAnt DICOM viewer, Medixant, Poland) and measurements were made on latero‐lateral radiographic views. During this reviewing and measuring, radiographs were only rejected if dorso‐ventral or cranio‐caudal obliquity deviated more than 10° from the latero‐lateral view. All radiographs in which the articular processes superimposed the intervertebral foramen by more than ∼50% were rejected. The intervertebral disc space was only measured when clearly visible. All decisions for inclusion or exclusion of radiographs in the study were made by an ECVDI board‐certified veterinary radiologist.

### 2.2 Data acquisition

**FIGURE 1:**
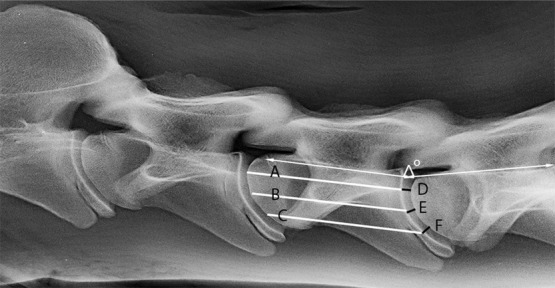
Latero‐lateral radiograph of the neck of an 18‐month‐old Dutch Warmblood horse. Equine Disc Width Index (EDWI) = (D+E+F)/(A+B+C) (white lines at vertebral body and black lines at intervertebral disc space). Normalized dorso‐ventral EDWI = (((D/B)/(F/B))/mean DHI) with mean EDWI = ((D/B)+(E/B)+(F/B)/3)). The vertebral alignment angle (VAA) at C4‐C5 is indicated by the white arrows. kVp 75, mAs 30, bone algorithm

**FIGURE 2:**
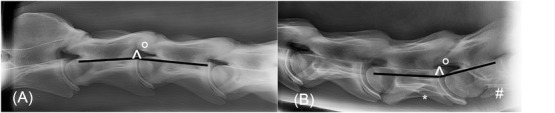
Latero‐lateral radiograph of the cranial part (A) and caudal part (B) of the neck of an 18‐month‐old Dutch Warmblood horse. A) Vertebral alignment angle at C2‐C3 was 188 degrees. B) Vertebral alignment angle at C6‐C7 was 172 degrees. Note the transposition of the caudal part of the transverse process from C6 (*) to C7(#). kVp 75–85, mAs 30–40, bone algorithm

The EDWI was calculated after measuring the intervertebral disc width (D+E+F) and vertebral length (A+B+C) of the cranial vertebra on three levels and defined as follows: EDWI = (D+E+F)/(A+B+C) (Figure 1,2).^17^ For measurement of the intervertebral disc space width of C2‐C3, the vertebral reference lines from C3 were used.

Measurements to calculate the EDWI of the horses at 1 month were made twice with a 2‐week interval by a fifth year veterinary student (DS) and a European College of Veterinary Diagnostic Imaging (ECVDI) board‐certified radiologist (S.V.). Measurements of the horses at 5 months old were made once by a fourth‐year veterinary student (R.dB.) and a board‐certified radiologist (S.V.), who also measured the horses once at 18 months old. The fourth year student (RdB) measured C2‐C3, C3‐C4, and C4‐C5 of the horses at 5 months old a second time 2 weeks after the first measurement.

A possible effect of the vertebral column curvature representing the head‐neck position (flexion, neutral, or extension) was evaluated by measuring the vertebral alignment angle (VAA) of the horses at 18 months (Figure 1). For this purpose, an alternative calculation of EDWI was introduced to create a so‐called normalized dorsal‐ventral DHI as described by Schmidt et al.^21^ This was defined as dorsal‐ventral EDWI = ((D/B)/(F/B)), mean EDWI being ((D/B)+(E/B)+(F/B)/3)) and the normalized dorsal‐ventral EDWI = (((D/B)/(F/B))/mean EDWI).^21^ The central vertebral reference line (B) was used in all calculations to rule out any difference between dorsal‐mid‐ventral measurements, except dorsal‐mid‐ventral intervertebral disc space width.

The presence or absence of congenital vertebral morphologic variation at C6 and C7 consisting of transposition of the transverse processes was recorded only in the 18‐month‐old horses.

### 2.3 Statistical analysis

Statistical analysis was performed by an ECVDI‐ board‐certified veterinary radiologist (S.V.) using commercially available software (SPSS version 24.0, IBM Statistics for Windows, USA) and a statistician (J.V.) using freely available software (R https://www.r-project.org/).^20^ The EDWI per intervertebral disc location was summarized as mean, standard deviation (SD), and range (min‐max). A Shapiro‐Wilk test was performed to evaluate for normal distribution of EDWI data hence parametric statistics applied for all except the effect of vertebral alignment angle on EDWI. For assessing the repeatability of the EDWI determination, the inter‐observer and intra‐observer variability at one month of age and inter‐observer variability at 5 months of age were evaluated by Bland‐Altman plots and the calculated intraclass correlation coefficient (ICC).

A linear mixed effect model^21^, ^22^ was used to analyze the association between the outcome of EDWI (measured by the radiologist) and location, age, and sex, and the interaction between location and age as explanatory factors. Horse was added to the model as random effect to take the correlation between repeated observations within a horse into account. Age was also used as random slope to account for the correlation between observations at different ages. The model was adjusted by a variance function to account for different variability per location. The model was reparametrized to compare the change of EDWI in course of time per location. The validity of the model was assessed by studying the residuals for normality and constant variance. The Akaike's Information Criterion was used to select the best model. A possible effect of angulation of the neck due to altering head‐neck position on EDWI was evaluated once at 18 months by applying Spearman correlation rank test between the vertebral alignment angle at 18 months and the normalized dorsal‐ventral EDWI at that age for all discs. Also, a possible effect of the presence of C6‐C7 transposition of the transverse processes on the EDWI at C6‐C7 was evaluated using an independent samples Student t‐test. For all tests, a *P*‐value of <.05 was considered significant.

## 3 RESULTS

The sample group consisted of n = 39 young Dutch Warmblood horses (Royal Dutch Sport Horses, n = 24 colts and n = 15 fillies). The young horses were sedated before the radiographic exams took place at 5 and 18 months with acepromazine (Neurotranq, Alfasan, Woerden, The Netherlands) (0.05 mg/kg bwt i.v.), and detomidine (Detogesic, Zoetis, Capelle a/d IIssel, The Netherlands) (0.01 mg/kg bwt i.v.). Right latero‐lateral radiographs were obtained (Fujifilm PcCR with 24 × 30 cm cassettes Fuijifilm Medical Systems France S.A.S., Steenbergen, The Netherlands; Philips S80, Philips Healthcare, Eindhoven, Netherlands) at settings of 55‐60 kV, 15‐20 mAs, and a focus‐film distance of 100 cm. The images were saved in DICOM format.

No intervertebral disc degeneration was presumed to be present because of the young age of the horses. During the 18‐month period a daily visual check of the total group was performed, and medical records were available for all young horses. None of the young horses had clinical signs related to the vertebral column, neither was any known to have suffered any form of trauma.

Including only the latero‐lateral views that were of sufficient quality resulted in a varying number of included foals per age and intervertebral disc space location. The number of foals ranged between eight (mostly C7‐Th1) and 35 foals and only these were included for further statistical analysis (Table 1). Measurements were mostly not possible at C7‐Th1 due to a reduced visibility of the intervertebral disc space by superimposed musculature of the fore limbs. The mean EDWI over all horses increased from cranial to caudal at the consecutive intervertebral disc spaces with a wide range at certain locations (Table 1). Bland‐Altman plots were created with the inclusion of limits of agreement (Supporting Information S2).

**TABLE 1:** Means and standard deviations for equine disc width index values recorded by three observers at three timepoints in sampled foals

	EDWI Veterinary Radiologist 1 month		EDWI Student DS 1 month		EDWI Veterinary Radiologist 5 months		EDWI Student RdB 5 months		EDWI Veterinary radiologist 18 months	
		Mean (SD)		Mean (SD)		Mean (SD)		Mean (SD)		Mean (SD)
Location	Number	min‐max	Number	min‐max	Number	min‐max	Number	min‐max	Number	min‐max
Overall	191	0.052 (0.011) 0.029 − 0.084	205	0.055 (0.011) 0.033 − 0.089	164	0.058 (0.011) 0.037 − 0.0895	165	0.056 (0.011) 0.033 − 0.086	150	0.052 (0.012) 0.032 − 0.088
C2‐C3	31	0.048 (0.007) 0.034 − 0.065	35	0.049 (0.009) 0.035 − 0.073	28	0.0498 (0.008) 0.039 − 0.075	30	0.047 (0.005) 0.034 − 0.061	29	0.042 (0.005) 0.033 − 0.053
C3‐C4	31	0.045 (0.007) 0.029 − 0.063	35	0.048 (0.009) 0.033 − 0.079	29	0.050 (0.008) 0.039 − 0.075	30	0.048 (0.005) 0.038 − 0.058	29	0.043 (0.006) 0.032 − 0.058
C4‐C5	33	0.047 (0.008) 0.036 − 0.065	35	0.051 (0.01) 0.034 − 0.089	29	0.054 (0.008) 0.037 − 0.067	31	0.053 (0.008) 0.033 − 0.065	29	0.049 (0.007) 0.037 − 0.062
C5‐C6	34	0.050 (0.008) 0.039 − 0.069	35	0.055 (0.009) 0.039 − 0.078	29	0.058 (0.007) 0.042 − 0.073	32	0.058 (0.006) 0.048 − 0.072	28	0.058 (0.008) 0.041 − 0.073
C6‐C7	32	0.057 (0.007) 0.045 − 0.073	35	0.062 (0.009) 0.046 − 0.079	30	0.067 (0.0098) 0.045 − 0.081	34	0.067 (0.009) 0.05 − 0.084	26	0.063 (0.011) 0.034 − 0.088
C7‐Th1	30	0.064 (0.009) 0.046 − 0.085	30	0.064 (0.009) 0.045 − 0.084	19	0.072 (0.008) 0.053 − 0.089	8	0.073 (0.008) 0.062 − 0.086	9	0.068 (0.009) 0.057 − 0.085

The EDWI (Equine Disc Width Index) is shown for the intervertebral disc spaces from C2‐C3 to C7‐Th1 separately and overall as mean with sd (standard deviation) and range from min (minimum) to max (maximum).

The bias within observers at one month is 0.0024 for the radiologist and −0.0004 for the student. The bias between observers was 0.0024 for the first measurements of radiologist and student and was 0.00026 for second measurements. At 5 months, the bias between measurements within observer (student) was −0.00053 and the bias between radiologist and student was −0.0012. The intra‐ and interobserver limits of agreement varied approximately between −0.01 and +0.01, except for the student at 5 months. Limits of agreement were approximately between 0.002 and −0.003 within observer. The ICC overall was 0.88 (Table 2) or even higher for both intra‐ and interobserver agreement regardless of location. Intraclass correlation coefficient per location varied between 0.78 and 0.98 for intra‐observer agreement and between 0.62 and 0.96 for interobserver agreement. Interobserver agreement was slightly better at C6‐C7 and C7‐Th1 for the second measurement than for the first at 1 month of age (Table 2).

**TABLE 2:** Intra‐ and inter‐observer intraclass correlation coefficients for Equine Disc Width Index values recorded by three observers at two time points in sampled foals

	Intra‐observer agreement						Inter‐observer agreement					
	Veterinary Radiologist 1 month		Student 1 month		Student 5 month		Veterinary Radiologist‐student 1 month, first measurement		Veterinary Radiologist‐ student 1 month, second measurement		Veterinary Radiologist‐student 5 months	
Location	Number	ICC (95%CI)	Number	ICC (95%CI)	Number	ICC (95%CI)	Number	ICC (95%CI)	Number	ICC (95%CI)	Number	ICC (95%CI)
Overall	177	0.91 (0.87−0.93)	205	0.95 (0.93−0.96)	56	0.95 (0.91−0.97)	186	0.88 (0.84−0.91)	178	0.91 (0.88−0.93)	149	0.94 (0.92−0.96)
C2‐C3	28	0.89 (0.78−0.95)	35	0.96 (0.91−0.98)	19	0.98 (0.94−0.99)	31	0.86 (0.70−0.93)	29	0.88 (0.72−0.94)	27	0.62 (0.20−0.83)
C3‐C4	29	0.85 (0.56−0.94)	35	0.96 (0.91−0.98)	19	0.98 (0.96−0.99)	31	0.78 (0.55−0.89)	30	0.84 (0.66−0.92)	28	0.69 (0.34−0.85)
C4‐C5	32	0.78 (0.37−0.91)	35	0.96 (0.92−0.98)	18	0.89 (0.73−0.96)	32	0.79 (0.56−0.89)	31	0.73 (0.45−0.87)	28	0.95 (0.89−0.98)
C5‐C6	31	0.89 (0.65−0.96)	35	0.86 (0.72−0.93)	NA	NA	33	0.82 (0.18−0.94)	31	0.78 (0.56−0.89)	28	0.89 (0.75−0.95)
C6‐C7	30	0.81 (0.51−0.92)	35	0.89 (0.79−0.95)	NA	NA	31	0.66 (0.10−0.86)	31	0.87 (0.73−0.94)	30	0.96 (0.91−0.98)
C7‐Th1	27	0.78 (0.52−0.89)	30	0.88 (0.75−0.94)	NA	NA	28	0.76 (0.47−0.89)	26	0.87 (0.71−0.94)	8	0.79 (0.10−0.96)

The ICC (intraclass correlation coefficients) with their CI (confidence interval) were recorded for intervertebral disc spaces C2‐C3 to C7‐Th1 and overall. NA = not available.

Results of the analysis to assess the association of EDWI with location in the neck and age are presented in Table 3. Sex was excluded for further analysis as inclusion did not contribute to the model fit. At 5 months of age, the mean EDWI at location C2‐C3 was slightly smaller than the mean EDWI at C3‐C4; C6‐C7 and C7‐Th1 were significantly larger than C3‐4. At 1 month, mean EDWI at all locations was not significantly smaller compared to 5 months of age. At 18 months, mean EDWI at C2‐C3 was larger, and mean EDWI at all other locations was smaller compared to the mean EDWI at the same location at 5 months of age, but not significantly. On average, the mean EDWI at 18 months at all locations except C2‐C3 was closer to the mean EDWI at 1 month than to the mean EDWI at 5 months.

**TABLE 3:** Parameter estimates from the regression model for the differences in mean Equine Disc Width Index per location in the neck for the sampled foals, related to age

Parameter	Estimate	95% Confidence interval	
Age 5 months			
LocationC2‐C3: age 5^2^	−0.0001	−0.0027	0.0025
InterceptC3‐C4: age 5^1^	0.0499	0.0475	0.0523
LocationC4‐C5: age 5^2^	0.0040	0.0013	0.0066
LocationC5‐C6: age 5^2^	0.0081	0.0055	0.0108
LocationC6‐C7: age 5^2^	0.0168	0.0136	0.0200
LocationC7‐T1: age 5^2^	0.0224	0.0183	0.0264
Compared with age 5 months			
Location C2‐C3:age 1^3^	−0.0011	−0.0037	0.0015
Location C3‐C4:age 1^3^	−0.0050	−0.0077	−0.0023
Location C4‐C5:age 1^3^	−0.0065	−0.0091	−0.0039
Location C5‐C6:age 1^3^	−0.0079	−0.0105	−0.0053
Location C6‐C7:age 1^3^	−0.0101	−0.0137	−0.0064
Location C7‐Th1:age 1^3^	−0.0087	−0.0133	−0.0040
Compared with age 5 months			
Location C2‐C3:age 18^3^	−0.0078	−0.0108	−0.0048
Location C3‐C4: age 18^3^	0.0066	−0.0097	−0.0035
Location C4‐C5:age 18^3^	−0.0051	−0.0082	−0.0021
Location C5‐C6:age 18^3^	−0.0002	−0.0033	0.0029
Location C6‐C7:age 18^3^	−0.0037	−0.0078	0.0005
Location C7‐Th1:age 18^3^	−0.0054	−0.0120	0.0012

EDWI, Equine Disc Width Index^1^ Estimated mean EDWI at the reference location C3‐C4 at age 5 month.

^2^ Estimated difference between mean EDWI at specified location and mean EDWI at C3‐C4 at 5 months of age ( = reference). ^3^ Estimated difference between mean EDWI at specified location and age (1 or 18 months) and mean EDWI at the same location at 5 months of age.

The mean vertebral alignment angle at 18 months varied between 175° at C5‐C6 and 190° at C2‐C3 (SD: 9°), while the normalized dorsal‐ventral EDWI had an overall mean of 25.4 (SD: 7.2). A low but significant correlation was found between the vertebral alignment angle and normalized dorsal‐ventral EDWI (ρ = 0.33, *P* < .0001; Table 4). There were 10 foals (10/28) that had recognizable transposition of the transverse processes at C6‐C7 at 18 months. The EDWI at 18 months between horses with or without transposition of the transverse processes did not differ significantly (Mean difference = −0.007, 95% confidence interval −0.024–0.01, *P* = .78).

**TABLE 4:** Means and ranges for vertebral alignment angle and normalized dorsal‐ventral Equine Disc Width Index values in sampled foals

	VAA (degrees)	Normalized DV‐EDWI
	Mean (min‐max)	Mean (min‐max)
Overall	180 (158−200)	25.4 (12.9−49.8)
C2‐C3	190 (181−200)	33.7 (21.4−49.8)
C3‐C4	186 (181−197)	24.3 (15.4−36.2)
C4‐C5	178 (172−190)	23.9 (15.7−39.7)
C5‐C6	175 (170−185)	21.4 (12.9−32.2)
C6‐C7	168 (158−177)	23.2 (13.95−42.6)

The VAA (vertebral alignment angle) in degrees and normalized DV‐EDWI (dorsal‐ventral equine disc width index) were recorded for intervertebral disc spaces C2‐C3 to C7‐C7 and overall as a mean and range from min(minimum) to max (maximum);.

## 4 DISCUSSION

This study aimed to describe intra‐ and interobserver agreement for the evaluation of equine cervical intervertebral disc space width by means of calculation of the equine disc width index (EDWI), a measurement previously termed disc height index (DHI) in other species than the horse.^15^, ^16^, ^17^ The outcome of this study presented initial reference values (mean ± 2SD; Supporting Information 1) for this new parameter in horses. It was based on measurements from a group of sound young horses, using a parameter with a good intra‐ and interobserver agreement. Interobserver agreement became even slightly better for the second measurement at one month of age at C6‐C7 and C7‐Th1 between the radiologist and student A, possibly indicating a learning effect.^22^ The number of available observations at C7‐Th1 was much lower at the age of 18 months primarily due to decreased visibility of the intervertebral disc as a result of superposition of soft tissues of the fore limbs. The EDWI was associated with a combination of the fixed factors “location” and “time,” showing an increase in mean EDWI at all locations except C2‐C3 between 1 and 5 months of age, but this increase had disappeared at 18 months except for the locations C5‐C6 and C6‐C7. This indicated that effectively there is no significant change over time of the EDWI from one to 18‐months however there is a temporary increase from 1 to 5 months and a decrease from 5 to 18 months of age. The increase at 5 months might possibly reflect a temporary disproportional growth with more rapid lengthening of the vertebral body at this age, whereas a slower vertebral growth pattern is present towards 18 months. Slowing down of the increase in withers height and body weight from 18 months onward could represent this slower growth rate after 18 months.^23^, ^24^ Although the cranial physeal closure occurs around two years and the caudal physis closes around 4 years,^25^ the EDWI reference values at 18 months of age might therefore be approaching the adult situation. The VAA also showed a low correlation with the normalized dorsal‐ventral EDWI. These results suggest that a standardized head‐ and neck position as well as sedating the horse may improve outcome when measuring the intervertebral disc space width, but further research is needed.

It has been described that the composition of the cervical intervertebral disc in horses differs from that in humans and companion animals.^11^, ^14^ In horses, the annulus fibrosis comprises the largest part of the intervertebral disc, whereas the nucleus pulposus is much smaller than in other species.^11^, ^14^ Narrowing of the intervertebral disc space due to the presence of C6‐C7 transposition of the transverse processes, congenital vertebral morphologic variation, degenerative herniation, or even discospondylitis with severe clinical signs in these horses has been described.^4^, ^5^, ^6^, ^7^, ^8^, ^18^, ^19^, ^26^ However, the intervertebral disc space width of horses without clinical signs has not been evaluated in these studies. Intervertebral disc degeneration is an ongoing process and has been described to start in humans during the first decade of life with molecular and structural changes such as loss of lamellar arrangement of the annulus fibrosis.^27^, ^28^, ^29^ Quantitative evaluation of the intervertebral disc space height has been implemented in humans.^29^ Radiographically determined DHI has proven to be a good parameter in human and animal studies to monitor the intervertebral space width during degeneration but also regeneration.^15^, ^16^, ^17^, ^31^, ^32^ As the cervical vertebral spine of the horse has a different morphology, curvature and shape, a similar DHI approach had to be evaluated first before this technique could be recommended for routine clinical application or development of measurement software. For this, one of the recorded DHI methods has been applied and creating the EDWI, with three reference lines in the cranial vertebral body and three measurements of the intervertebral disc space width.^17^ This method was preferred over others, as the head‐neck position has a large effect on the curvature and hence the intervertebral alignment of the long and flexible equine neck; there are no such effects on the far more rigid thoracolumbar column.^15^, ^16^, ^31^ This approach of determining the EDWI was shown to be a repeatable method in young horses. The agreement interval was moderately wide with limits of agreement were between around ‐0.01 and +0.01. Therefore, some reservations are needed at this stage, as criteria for what is biologically acceptable are not known yet in horses. Future establishment of EDWI reference values for the adult non‐degenerated and degenerated equine intervertebral disc is needed. As the composition of the equine intervertebral disc is slightly different from other species, degeneration has possibly a different effect on intervertebral disc space width compared to species with a less fibrous nucleus pulposus such as dogs and humans. The macroscopic yellow discoloration, fibrillation and cleft formation as described in the horse^14^ could be a pre‐stage of collapse, as seen in end‐stage discospondylitis.^18^, ^19^

Limitations of this study include the fact that measurements were performed in young horses up to 18 months, but not in adult horses and the sample size was relatively small. Increased DHI due to widening of the radiographic intervertebral disc space has been reported as part of disc regeneration.^31^ Widening of the dorsal part of the intervertebral disc space during flexion or ventral part of the intervertebral disc space in the equine neck during extension could alter EDWI as well, as evidenced by the significant association between the vertebral alignment angle and normalized dorsal‐ventral DHI. In this study, there was no standardized head and neck position with variation present in head height and latero‐lateral angulation. Although standardization would be preferred, most standard measurements in the equine cervical spine (such as minimal sagittal diameter, intravertebral sagittal diameter ratio and vertebral fossa angle) have been found to be equal or only minimally different in neutral versus low or high neck position.^32^ Therefore, some margin for error in positioning horses for radiographs of the neck may be acceptable. Further investigation and evaluation of positioning effects on EDWI should be considered.

In this study, the assumption was made that foals or young horses are not prone to disc degeneration yet, but we could not fully exclude occurrence of an unnoted traumatic insult, as the foals were kept in the fields as a group. Occurrence of any substantial form of trauma was deemed highly unlikely, however, as none of the foals exhibited any clinical signs related to the vertebral column during the course of the study.

In conclusion, findings indicated that EDWI is a reliable and repeatable radiographic method for quantifying cervical intervertebral disc space width in young horses. Findings also provided reference values for EDWI in clinically normal young horses that can be used as background in future studies. The EDWI did not alter significantly in time per location in young horses up to 18 months; however, a growth peak at 5 months was noted. Authors recommend that the head‐neck position be standardized in future studies when determining the EDWI with head height just above the level of the shoulders. Further evaluation of this method is also needed in a larger population of adult warmblood horses with the concomitant degenerative status of the intervertebral discs known.

## LIST OF AUTHOR CONTRIBUTION

### Category 1

(a) Conception and Design: Veraa, Scheffer, Back
(b) Acquisition of Data: Veraa, Scheffer, Smeets, de Bruin, Hoogendoorn
(c) Analysis and Interpretation of Data: Veraa, Smeets, de Bruin, Vernooij, Nielen

### Category 2

(a) Drafting the Article: Veraa, Vernooij, Nielen
(b) Revising Article for Intellectual Content: Veraa, Scheffer, Smeets, de Bruin, Vernooij, Nielen, Hoogendoorn, Back

### Category 3

(a) Final Approval of the Completed Article: Veraa, Scheffer, Smeets, de Bruin, Vernooij, Nielen, Hoogendoorn, Back

## Supporting information

Supporting Information

Supporting Information

Supporting Information

Supporting Information

Supporting Information

Supporting Information

Supporting Information
