# Supplementary material for: Cervical disc width index is a reliable parameter and consistent in young growing Dutch Warmblood horses
Source: Vet Radiol Ultrasound. 2020 Oct 13;62(1):11–9. doi: 10.1111/vru.12913 (PMC7894175; doi:10.1111/vru.12913)
Supplement: Supplementary file 1 — Supporting Information [file VRU-62-11-s001.docx]

Supplementary item 1: Overview of the reference values (mean ±2 standard deviation) at 1, 5 and 18 months of age for the different observers.

| Location | DHI Radiologist 1 month | DHI Radiologist 5 months | DHI radiologist 18 months | DHI Student DS 1 month | DHI Student RdB 5 months |
| --- | --- | --- | --- | --- | --- |
|  | Mean ±2 sd | Mean ±2 sd | Mean ±2 sd | Mean ±2 sd | Mean ±2 sd |
| Overall | 0.030-0.074 | 0.036-0.080 | 0.028-0.076 | 0.033-0.077 | 0.034-0.078 |
| C2-C3 | 0.034-0.062 | 0.034-0.066 | 0.032-0.052 | 0.031-0.067 | 0.037-0.057 |
| C3-C4 | 0.031-0.059 | 0.034-0.066 | 0.031-0.055 | 0.030-0.066 | 0.038-0.058 |
| C4-C5 | 0.031-0.063 | 0.038-0.070 | 0.035-0.063 | 0.031-0.071 | 0.037-0.069 |
| C5-C6 | 0.034-0.066 | 0.044-0.072 | 0.042-0.074 | 0.039-0.073 | 0.046-0.070 |
| C6-C7 | 0.043-0.071 | 0.047-0.087 | 0.041-0.085 | 0.044-0.080 | 0.049-0.085 |
| C7-Th1 | 0.046-0.082 | 0.056-0.088 | 0.050-0.086 | 0.046- 0.082 | 0.057-0.089 |

Supplementary item 2: Bland-Altmann plots for intra-observer and inter-observer agreement. The mean ± 2sd reference lines are provided as is the linear slope. Intra-observer a) for radiologist at one month (mean of difference 0.0024, standard deviation 0.005), b) for student DS at one month (mean of difference -0.0004, standard deviation 0.005), c) for student RdB at five months (mean of difference -0.0005, standard deviation 0.001); and interobserver d) for radiologist versus student DS at one month first measurement (mean of difference 0.0024, standard deviation 0.007), e) for radiologist versus student DS at one month second measurement (mean of difference 0.0003, standard deviation 0.006), f) for radiologist versus student RdB at five months (mean of difference -0.0012, standard deviation 0.005).
