# Supplementary figures and images for: Cervical disc width index is a reliable parameter and consistent in young growing Dutch Warmblood horses
Source: Vet Radiol Ultrasound. 2020 Oct 13;62(1):11–9. doi: 10.1111/vru.12913 (PMC7894175; doi:10.1111/vru.12913)

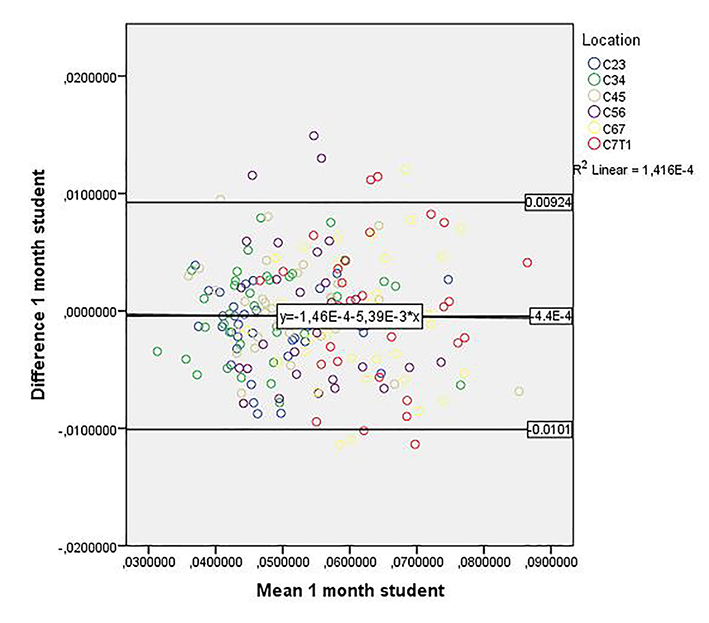

Supplement: Supplementary file 2 — Supporting Information [file VRU-62-11-s002.tif]

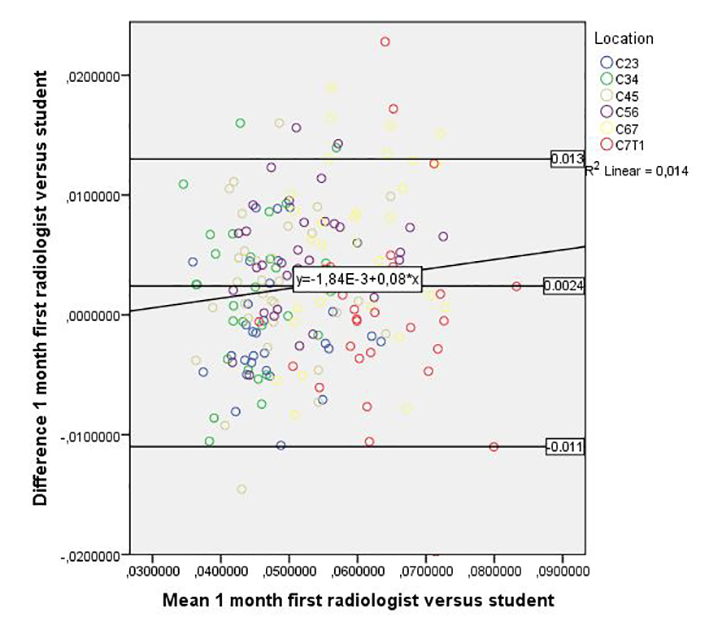

Supplement: Supplementary file 3 — Supporting Information [file VRU-62-11-s003.tif]

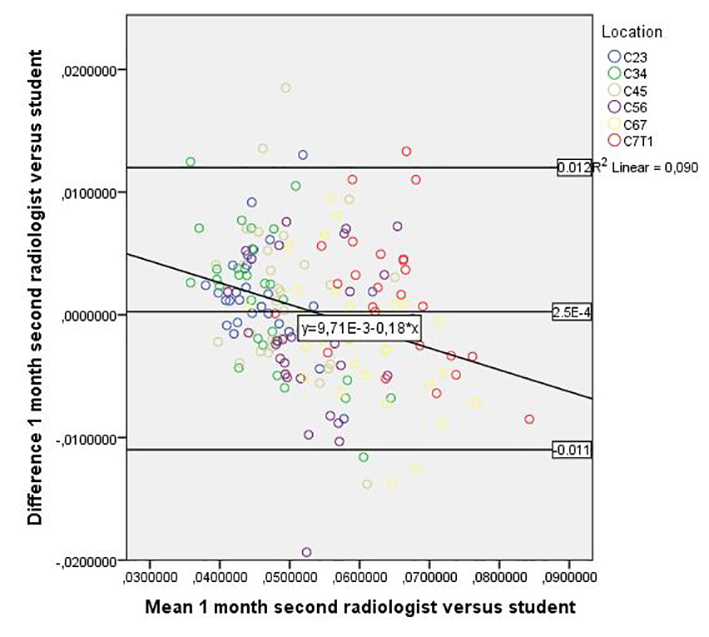

Supplement: Supplementary file 4 — Supporting Information [file VRU-62-11-s004.tif]

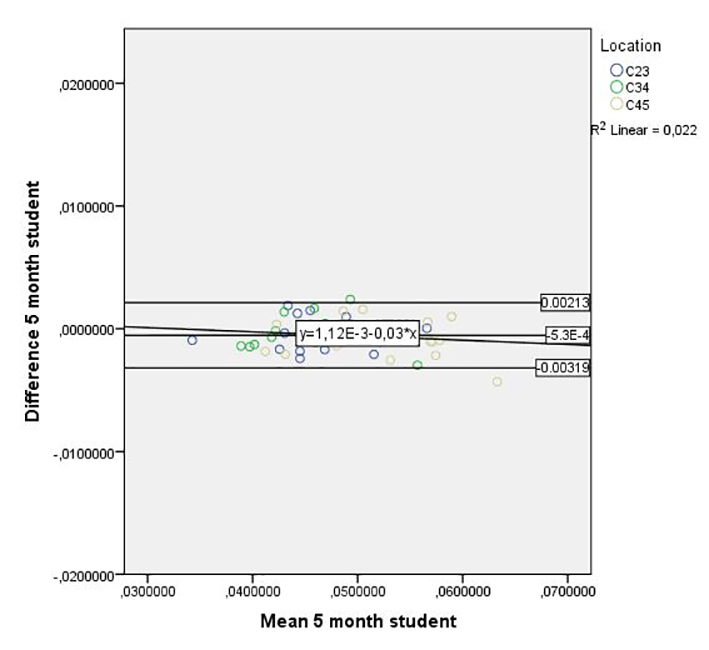

Supplement: Supplementary file 5 — Supporting Information [file VRU-62-11-s005.tif]

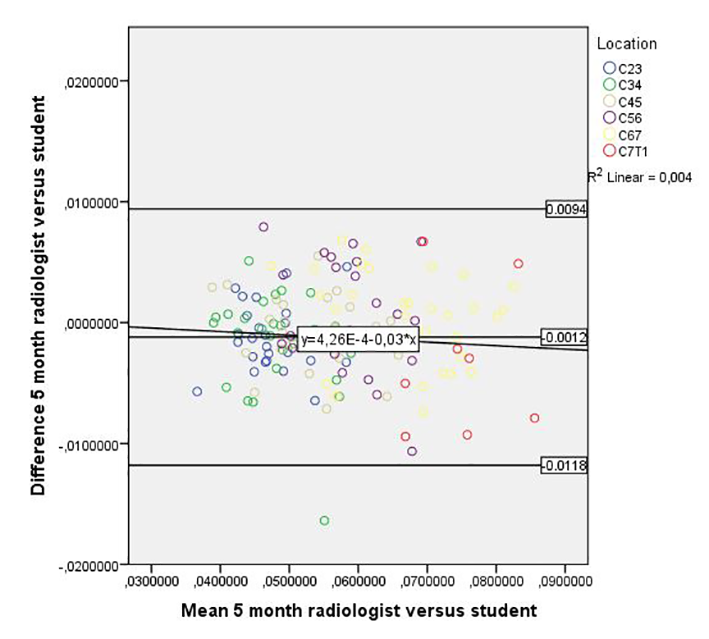

Supplement: Supplementary file 6 — Supporting Information [file VRU-62-11-s006.tif]

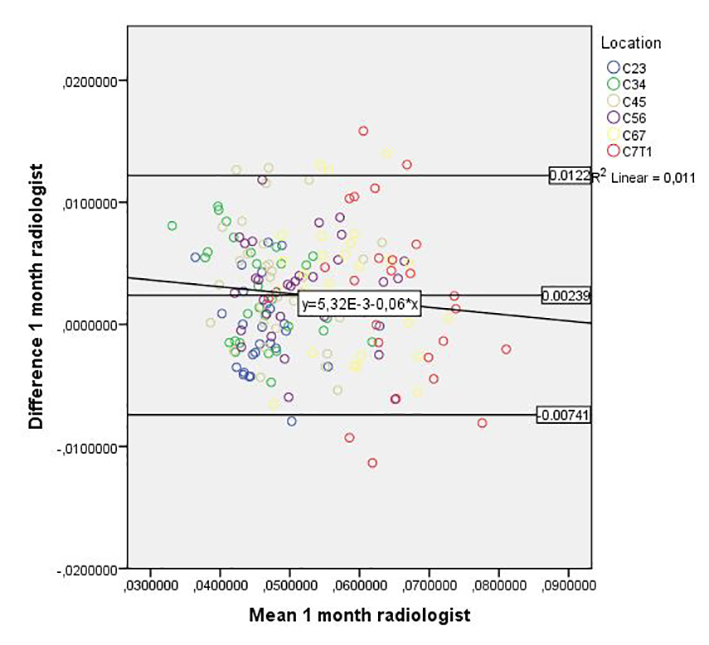

Supplement: Supplementary file 7 — Supporting Information [file VRU-62-11-s007.tif]
